# Supplementary material for: Identification of photoperiod-induced specific miRNAs in the adrenal glands of Sunite sheep (Ovis aries)
Source: Front Vet Sci. 2022 Jul 22;9:888207. doi: 10.3389/fvets.2022.888207 (PMC9354845; doi:10.3389/fvets.2022.888207)
Supplement: Supplementary file 1 [file Data_Sheet_1.ZIP › Supplementary file/miRDeep2 file.zip!/miRDeep2 file/Know_Structure/SPLP42A2/oar-let-7g.pdf]

miRBase precursor : oar-let-7g
Total read count : 745357
oar-let-7g read count : 745338
remaining reads : 19

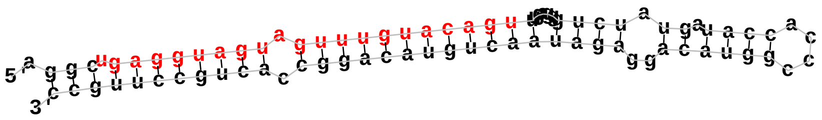

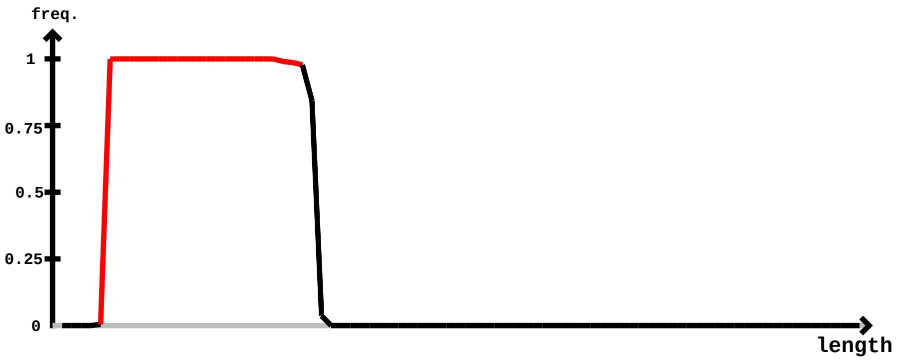


```
      oar-let-7g
5'- aggcugagguaguaguuuguacaguuugagggucuaugauaccacccgguacaggagauaacuguacaggccacugccuugcc   -3'    exp
    .(((.((((((((.((((((((((((.....((((.((.((((....))))))..))))))))))))))))).)))))))))))    reads      mm        sample
    ...cugagguaguaguuugu...............................................................    2          0         seq
    ...cugagguaguaguuuguac.............................................................    48         0         seq
    ...cugagguaguaguuuguaca............................................................    17         0         seq
    ...cugagguaguaguuuguacag...........................................................    41         0         seq
    ...cugagguaguaguuuguacagu..........................................................    702        0         seq
    ...cugagguaguaguuuguacaguu.........................................................    2539       0         seq
    ...cugagguaguaguuuguacaguuu........................................................    98         0         seq
    ....ugagguaguaguuugua..............................................................    326        0         seq
    ....ugaAguaguaguuugua..............................................................    1          1         seq
    ....ugagguaAuaguuugua..............................................................    1          1         seq
    ....ugagguagCaguuugua..............................................................    1          1         seq
    ....ugagguaguaguuuguac.............................................................    6741       0         seq
    ....ugagguagCaguuuguac.............................................................    9          1         seq
    ....ugagguaguaguuuguaca............................................................    3160       0         seq
    ....ugagguaguaguuuguacag...........................................................    6278       0         seq
    ....ugagguaguaguuuguacagu..........................................................    99078      0         seq
    ....ugagguaguaguuuguacaguu.........................................................    598575     0         seq
    ....ugagguaguaguuuguacaguuu........................................................    27219      0         seq
    ....ugagguaguaguuuguacaguuug.......................................................    18         0         seq
    ....ugagguaguaguuuguacaguuuga......................................................    1          0         seq
    .....gagguaguaguuuguac.............................................................    5          0         seq
    .....gagguaguaguuuguaca............................................................    3          0         seq
    .....gagguaguaguuuguacag...........................................................    5          0         seq
    .....gagguaguaguuuguacagu..........................................................    45         0         seq
    .....gagguaguaguuuguacaguu.........................................................    398        0         seq
    .....gagguaguaguuuguacaguuu........................................................    20         0         seq
    ......agguaguaguuuguacagu..........................................................    1          0         seq
    ......agguaguaguuuguacaguu.........................................................    6          0         seq
    .........................................................auaacuguacaggccacugccuu...    1          0         seq
    ...........................................................aacuguacaggccacugccuugc.    1          0         seq
    ............................................................acuguacaggccacugccuugc.    1          0         seq
    .............................................................cuguacaggccacugccuugc.    9          0         seq
    .............................................................cuguacaggccacugccuugcc    7          0         seq
```
